# Expectation or Sensorial Reality? An Empirical Investigation of the Biodynamic Calendar for Wine Drinkers

**DOI:** 10.1371/journal.pone.0169257

**Published:** 2017-01-03

**Authors:** Wendy V. Parr, Dominique Valentin, Phil Reedman, Claire Grose, James A. Green

**Affiliations:** 1 AGLS Faculty, Lincoln University, Christchurch, New Zealand; 2 Centre des Sciences du Goût et de l’Alimentation. CNRS, INRA, Université de Bourgogne Franche Comté, Dijon, France; 3 Phil Reedman, Adelaide, Australia; 4 Institute of Plant and Food Research, Marlborough, New Zealand; 5 University of Otago, Dunedin, New Zealand; Oregon Health and Science University, UNITED STATES

## Abstract

The study’s aim was to investigate a central tenet of biodynamic philosophy as applied to wine tasting, namely that wines taste different in systematic ways on days determined by the lunar cycle. Nineteen New Zealand wine professionals tasted blind 12 Pinot noir wines at times determined within the biodynamic calendar for wine drinkers as being favourable (Fruit day) and unfavourable (Root day) for wine tasting. Tasters rated each wine four times, twice on a Fruit day and twice on a Root day, using 20 experimenter-provided descriptors. Wine descriptors spanned a range of varietal-relevant aroma, taste, and mouthfeel characteristics, and were selected with the aim of elucidating both qualitative and quantitative aspects of each wine’s perceived aromatic, taste, and structural aspects including overall wine quality and liking. A post-experimental questionnaire was completed by each participant to determine their degree of knowledge about the purpose of the study, and their awareness of the existence of the biodynamic wine drinkers’ calendar. Basic wine physico-chemical parameters were determined for the wines tasted on each of a Fruit day and a Root day. Results demonstrated that the wines were judged differentially on all attributes measured although type of day as determined by the biodynamic calendar for wine drinkers did not influence systematically any of the wine characteristics evaluated. The findings highlight the importance of testing experimentally practices that are based on anecdotal evidence but that lend themselves to empirical investigation.

## Introduction

Influence of extrinsic factors on perceived liking and quality of foods and beverages is now well established, with research demonstrating effects from a variety of stimuli including ambient music, consumption location (e.g., a laboratory vs a restaurant) and wine bottle shape [1]. The present study investigated a contentious factor, namely the impact of the lunar cycle on perception of Pinot noir wine qualities by experienced wine professionals.

Over recent decades the wider agricultural philosophy of biodynamics, founded by Austrian philosopher Rudolf Steiner in the 1920s [2], has systematically increased in influence within the international wine industry. This is exemplified by recent articles in media such as *The Drinks Business* [3] and *The Independent* [4]. Conceivably, a reason for the current interest in biodynamic practice relates to a desire by wine producers to farm more sustainably, with focus on environmental issues such as soil health in keeping with general societal trends. Recently there has been extension of biodynamic philosophy from viticultural practice and wine production to include wine tasting [5]. The aim of the present study was to investigate a central tenet of biodynamic philosophy as applied to wine tasting, namely that wines taste different in systematic ways on days determined by the lunar cycle.

Biodynamic practices have their basis in a series of lectures titled *Spiritual foundations for a renewal of agriculture* [2]. Biodynamic agriculture follows the central tenets of organic production, namely exclusion of synthetic fertilisers and chemical herbicides that are common in conventional agriculture, and inclusion of practices such as mulching, manure and composts, but adds further elements. These include focus on lunar rhythms, self-sufficiency, and the use of specially prepared sprays and composts that involve cow manure. There is much scepticism amongst scientists about biodynamics as a form of agriculture, this lack of scientific respectability presumably, at least in part, due to the relative lack of sound empirical data on the topic. The dearth of sound data in turn is understandable in that many of the beliefs and practices of biodynamics do not lend themselves easily to empirical testing. Further, it is virtually impossible to control the myriad of potentially confounding factors when making comparisons of viticultural and/or oenological practice as a function of farming type (conventional; organic; biodynamic). Despite these difficulties, a longitudinal study over 21 years that compared biodynamic, bioorganic, and conventional farming practices in Switzerland [6] reported enhanced soil fertility and increased biodiversity in their organic plots relative to the conventionally farmed plots. However, in a recent viticultural study [7], fruit quality was reported as not influenced by the organic and biodynamic management systems investigated. In terms of wine production, one of the few studies comparing biodynamic and organic viticulture that has been published in a peer-reviewed journal reported no consistent differences in any of the physical, chemical, and biological parameters measured [8]. To our knowledge there are no wine sensory data in support of advantages of biodynamic practices over conventional farming practices in terms of enhanced sensory experience although a recent investigation reported on this topic indirectly. When over 70,000 wine assessments (wine ratings) given to wines in three publications, Wine Advocate, Wine Enthusiast, and Wine Spectator, were analysed [9], the authors concluded that organic or eco-certification, the latter including organic and biodynamically-produced wines, was statistically associated with increased wine quality ratings in red wines, but not in white wines.

The biodynamic calendar for wine drinkers was first produced in German approximately fifty years’ ago by Maria Thun and is now published by her son Matthias Thun [5]. Since 2010, the calendar has been published annually in the English language for the United Kingdom time zone, namely for GMT/British Summer time, by Floris Books in Edinburgh and is available also in ‘phone App form. The iPhone App called ‘Wine Tonight’ not only provides UK consumers with information as to whether it is considered a favourable day or unfavourable day to drink their wine but as well offers conversion to other time zones including those of southern hemisphere countries.

The wine-drinking advice in the calendar is based on biodynamic farming principles, a key tenet being that many agricultural practices are timed according to the moon’s cycle. The calendar advises wine drinkers accordingly; that is, the calendar provides ‘days’ when the moon’s rhythms suggest that a wine will taste its best. The ‘days’ are seldom 24 hour periods but are temporal intervals categorised according to star constellations and the movement of the moon in terms of ascending and descending cycles. Four types of days have been identified [5] based on when the moon moves through the twelve constellations. When the moon moves into Sagittarius, Aries, and Leo a Fruit day takes over; when in Libra, Aquarius, and Gemini, a Flower day occurs; Leaf days involve the moon moving through Scorpio, Pisces, and Cancer while Root days take over when the moon moves into Virgo, Capricorn and Taurus. Fruit and Flower days are considered favourable for wine tasting while Leaf and Root days are best avoided.

To our knowledge, there are no published, sound empirical data to support the notion that wines taste different according to where the moon is in its lunar cycle. None-the-less, the notion that the moon’s rhythms exert influence on the taste of a wine in systematic ways does lend itself to empirical investigation. Anecdotal evidence in the form of wine industry media [10] suggests that some professionals in the wine industry, in particular wine producers and retail outlet and wine distribution company staff, appear to accept that the moon may exert some sort of influence over how a beverage tastes on a particular day, despite the lack of scientific evidence. For example, in the United Kingdom, several major supermarkets and wine retail outlets such as Tesco and Marks & Spencer have been reported as organising their wine tasting sessions around "good" days (Flower days; Fruit days) and "bad" days (Leaf days; Root days) as dictated by the lunar calendar [10] [11] [5]. Published anecdotal reports provide some details as to precisely how wines are expected to change in terms of being ‘better’ or ‘worse’ as a function of the lunar cycle such as becoming more tannic or bitter on Leaf days and Root days, the days that are argued as not favourable for wine tasting, while expressing better their freshness and aromatic qualities on Fruit days and Flower days, days argued as best for wine tasting [12] [13].

It is conceivable that respect within the wine industry for the notion that the moon’s rhythms influence a wine’s taste has its basis in attempts to understand why some wines do appear to taste differently across different days. Anecdotal evidence suggests that it is not unusual for wine professionals and knowledgeable wine consumers to report that a wine, including a bottled wine, appears to taste “different” when tasted at varying time periods such as on consecutive days or weeks. For example, a wine may be perceived as tasting different across two successive tastings of the same wine, or “not showing well” on a particular day [14]. There are many reasons that could underlie such perceived differences including wine composition factors, weather and atmospheric pressure, and human perception factors including memory and mood of the taster. Hence, the potential factor investigated in the present study is one possibility only, but one that does lend itself to empirical investigation.

One convoluted notion to justify why the moon may influence the taste of wine is based on the known impact of the lunar cycle on the tides. This has been interpreted and extended by followers of biodynamic philosophies to argue that the moon therefore also affects the water in plants, the water in a wine, and the water in the human body. The latter notion includes the argument that human behaviour (e.g., moods) is affected by the lunar cycle such that human sensory experience including the tasting of wine is influenced by the moon. Hence, there are broadly two possibilities: a wine may change (e.g., in some aspects of composition such as bonding of chemical compounds) according to the lunar cycle, or the taster may perceive the wine to change. In the present study we separate these two aspects by providing sensory data addressing the question “are the wines reported by experienced wine professionals as tasting better on Fruit days in comparison with Root days?”, as well as providing wine composition data where data analysis was conducted on the same wines employed in the sensory study on the same day on which they were tasted (i.e., on both a Fruit day and on a Root day).

To test the notion that wines taste different in systematic ways on days determined by the biodynamic wine drinkers’ calendar [5], several aspects of methodology required consideration. First, it was essential that all wine tasters were blind as to the purpose of the study and in terms of following the biodynamic wine drinkers’ calendar. To determine this, each wine professional completed a questionnaire after all tasters had participated in the study, the questionnaire seeking the relevant information [see S1 Questionnaire]. A second and very important factor was to effect a correct time-zone change to ensure that our selected Fruit days and Root days were valid in terms of the ascending and descending moon cycle. This was achieved by consultation with several relevant people and their publications (e.g., the Astro-Calendar produced by Brian Keats in Tasmania) [15], and confirming that the conversions offered for New Zealand Summer Time matched the conversion available via the iPhone App associated with the calendar for the United Kingdom time zone produced by Floris Books (www.florisbooks.co.uk) [5]. A third important factor was to operationalise *different* in terms of its application to how the wines would be expected to taste on a good day (Fruit day) and on a less favourable day (Root day) according to the calendar. Anecdotal evidence from online, published reports [12], along with interview data from UK wine-industry professionals purporting to use the wine drinkers’ calendar, was employed to determine the wine type best suited to testing our hypothesis, along with the wine characteristics most likely to serve as appropriate descriptors. Pinot noir was selected as the wine of choice. This was due to its varietal characteristics, namely an aromatic profile that could be more-or-less expressive, and a tannin profile that could range between silkiness and harshness [16], these aspects of wine sensory experience involving the predominant attributes reported within the anecdotal evidence as changing according to the lunar cycle. Third, we selected relatively young wines for the study given that according to the biodynamic calendar [5] a wine greater than five years of age may be advantaged by being tasted on a Leaf day rather than on a Fruit or Flower day. Fourth, we selected wines from biodynamic, organic and conventional wine producers to comprise the sample set for the study as the lunar cycles’ influences are argued as relevant irrespective of wine-production mode [5]. Finally, since several media references made to tasting according to biodynamic philosophy [17] have suggested that weather patterns (e.g., atmospheric pressure) may also influence how a wine tastes, we collected relevant meteorological data. During the time that the experimental tastings were conducted, we recorded mean per hour measures of moisture (rain in mm), sunshine (minutes), wind speed, and atmospheric pressure.

### Summary and hypothesis

In the current study our experimental hypothesis was that Pinot noir wines would be reported as tasting different in systematic ways on days determined by the biodynamic calendar for wine drinkers [5]. More specifically, we predicted that the wines would be perceived as more aromatic, fruity, concentrated, and overall flavoursome on Fruit days than on Root days. Conversely, wines were predicted to be perceived on a Root day as less balanced, more aggressive in terms of tannin influences, and with any green or leafy characteristics, over-oaking, or faults (e.g., reductive phenomena) becoming dominant.

## Methods and Materials

### Participants

Nineteen New Zealand (NZ) wine professionals participated in the study. All participants were experienced with production and tasting of Pinot noir wines. They were members of a panel of wine tasters who regularly participate in wine sensory research tastings and are involved in various types of wine production methods including conventional, organic and biodynamic. Mean age of the participants was 41.5 years (age range = 29–60 years), and there were 5 females and 14 males. The majority of participants were oenologists, winemakers and wine producers (*N* = 16), one reported her major occupation as viticulturist, and two participants were wine science educators. Seven of the participants were also formally designated wine judges. Two participants only reported that they were smokers. Mean number of years of wine industry experience was 18.2 years (range = 8–32 years). The experiment was performed in keeping with ethical requirements of the Lincoln University Human Ethics Committee, NZ, with informed written consent obtained prior to participation. Participants were ‘blind’ as to the purpose of the tasting, with experimenter-provided instructions advising them only that the wine varietal under evaluation was Pinot noir, and that they would need to attend two separate tasting sessions.

### Materials

Twelve Pinot noir wines from NZ’s major Pinot noir producing areas were selected for the experiment (Table 1). Eight wines were from the 2012 vintage and 4 were from the 2013 vintage. The wines were from conventional, organic, and biodynamic producers. Wines were sourced directly from their producers, and all were sealed with screw-cap closure to ensure consistency between bottles and between tasting days. The wines, listed in Table 1, comprised four wines from Marlborough, three from Central Otago, two from each of Martinborough and Nelson, and one wine from Canterbury (Waipara). All wines were 100% Pinot noir and ranged in price between NZ$30 - $50. The wines were selected by senior researchers and wine professionals on the basis of three criteria. These criteria were that each wine was judged by its producers as (i) exhibiting Pinot noir varietal fruity characters; (ii) comprising a phenolic (tannin) composition that provided perceived substance in terms of wine in-mouth structure, and (iii) being relatively youthful (younger than three years of age). The wines were stored at 14^0^ C until 24 hours before a session at which time they were slowly brought up to ambient temperature (22^0^ C, + or– 1).

**Table 1:** Pinot noir wines employed in the study.

Wine	Year	Alcohol v/v	Region of NZ	Production method
LDHR	2013	13.6	Marlborough	Conventional
ASB	2012	13.0	Marlborough	Conventional
CHPN	2013	13.7	Marlborough	Organic
HPN	2012	13.3	Marlborough	Organic
MDPN	2012	13.4	Central Otago	Conventional
APN	2012	14.1	Central Otago	Organic
QRPN	2012	14.0	Central Otago	Biodynamic
WWW	2013	14.1	Waipara	Conventional
PPPN	2012	12.7	Martinborough	Conventional
MVTT	2013	13.8	Martinborough	Conventional
WNPN	2012	13.1	Nelson	Organic
NMPN	2012	13.1	Nelson	Conventional

### Procedure

#### Sensory study

The study was conducted at the sensory facilities of the Marlborough Wine Research Centre (MWRC) in Blenheim, NZ. The specialised sensory facilities at MWRC permitted the important variables such as ambient temperature, sound, ambient odours and between-participant communication to be controlled as advised for sensory experimentation [18]. Each taster participated in two sessions separated by approximately one week, one session on a Fruit day and a second session on a Root day, these ‘days’ determined by the biodynamic calendar for wine tasting in 2014 [5]. It was not possible to control strictly the temporal gap between the two tastings per person but the time between each person’s two sessions ranged between 7–9 days. Four to nine people participated at any particular time, and all tastings were conducted between the hours of 1 pm and 7 pm on a tasting day. Twelve participants undertook their first session on a Fruit day and their second session on a Root day. The remaining seven participants tasted the wines in the reverse order; that is, their first session was conducted on a Root day and their second session on a Fruit day. Each session lasted approximately two hours.

The wines for evaluation comprised 25-mL samples that were served at ambient temperature in standardised tasting glasses [19]. A new bottle of each wine was opened each day that the experiment was conducted and the wines were first checked for faults by two experienced wine professionals. For the within-session, replicate data collection, the wines were re-poured between flight 1 and flight 2 for each participant. The glasses were coded with 3-digit numbers and were covered with plastic Petri dishes. Each participant evaluated the 12 wines within the sample set four times, twice in each session with the two within-session tastings separated by a 20-minte break. Order of the 12 wines within a flight was varied between-subject but remained constant within-subject. That is, the wine samples were presented in a different order specific to each participant according to a Williams Latin square arrangement generated by FIZZ software (Biosystemes, Courtenon, France). On the other hand, the wine order specific to any particular participant remained the same for each of the participant’s four tastings of the 12 wines to eliminate any possibility of wine order as a confounding effect across Fruit and Root day tastings. Water was available throughout each session.

Participants were seated in separate booths or at separate tables where their 12 wines were positioned. They were advised that they were to undertake two tasting tasks within the session, and that they could proceed with the tasks at their own pace. Participants were also advised that they were welcome to take a break at any time should they choose such, but that they must take a 20-minute break between the two descriptive rating tasks within the session. Specific instructions to participants prior to each task included that they were to evaluate each wine, in the order presented, via 20 experimenter-provided descriptors. They were also informed that the wine evaluations were to be undertaken by global perception, that is, by full tasting involving olfaction, taste, and trigeminal stimulation. They were further advised that all wine was to be expectorated (i.e., not swallowed). The 20 descriptors (see Table 2) were presented in the same order for every participant and for all four descriptive rating tasks performed by each participant. The descriptors spanned a range of varietal-relevant aroma, taste, and mouthfeel characteristics, and were selected with the aim of elucidating both qualitative and quantitative aspects of each wine’s aromatic (e.g., fruity) and more structural aspects (e.g., balance; length in mouth; harshness of tannins). There were eight Intensity Descriptors (aromatic intensity; fruit notes; green notes; reductive notes; concentration in mouth; bitterness; astringency; sweetness), six Quality Evaluation descriptors (overall quality; oak integration; acid/flavour balance; harmony of components; overall structure; length in mouth), four Qualitative descriptors (expressiveness; fruit ripeness; tannins; colour) and finally two Overall Appreciation descriptors (Pinot noir typicality; liking). Each descriptor was rated via a 100 mm, horizontal visual analogue scale with the scale anchors as in Table 2.

**Table 2:** The 20 descriptors employed in the experiment in the order presented.

Descriptors	Scale anchors
**Intensity descriptors**	
Aromatic intensity	Low—intense
Fruit notes	Low—intense
Green notes	Low—intense
Reductive notes	Low—Intense
Concentration in mouth	Low—intense
Bitterness	Low - Intense
Astringency	Low - Intense
Sweetness	Low - Intense
**Quality evaluation**	** **
Overall quality	Poor—Outstanding
Oak integration	Poor—Outstanding
Acid/Flavour balance	Poor—Outstanding
Harmony of components	Poor—Outstanding
Overall structure	Poor—Outstanding
Length in mouth	Poor–Outstanding
**Qualitative descriptors**	
Expressiveness	Closed—Expressive
Fruit ripeness	Unripe–Raisined
Tannins	Harsh–Soft
Colour	Light—Dark
**Overall appreciation**	
Pinot noir typicality	Atypical—Typical
Liking	Dislike—Like

#### Post-experiment questionnaire

After all 19 participants had completed both sessions of the experiment, each participant was sent a Questionnaire [S1 Questionnaire] to determine their degree of knowledge about the purpose of the study, and their awareness of the existence of the wine drinkers’ calendar [5].

#### Wine basic parameters

At the time of the sensory sessions on both a Fruit day and a Root day, wine samples were taken for physico-chemical analysis of standard wine parameters. The wine parameters were determined by InfraRed spectrometry using Fourier Transformation (IRFT) with a Winescan^TM^ FT2 (FOSS) that was calibrated with wine samples analysed in accordance with official OIV practices. Samples were analysed in duplicate and parameters were quantified using a high-input calibration file. Relative standard deviations were exclusively lower than 10%.

#### Collection of meteorological information

Hourly measures of air pressure (hPa) for Blenheim were recorded from the NZ Meteorological Service website (www.metservice.com) during each tasting session, along with basic meteorological data including rainfall, sunshine minutes, relative humidity, and wine speed.

### Data Analysis

Due to the controversial nature of the topic under investigation, the data analysis was performed ‘blind’. That is, the data were analysed by a research colleague who was unaware of the purpose of the study, not involved in any aspects of planning or implementation of the experiment, and received the dated coded so that the study’s variables were not apparent (e.g., Fruit day data were coded as Variable X, and Root day data were coded as Variable Y).

### Sensory data

To consider the effect of Fruit versus Root days, mixed effects ANOVAs were computed for each descriptor with condition (Fruit day; Root day) and replicate as fixed factors, and with wine and subject as random factors, along with an interaction term between condition and replicate. A significance level of 0.1 was adopted for all analyses to ensure that we did not miss any potential effects, even though this is a more liberal criterion than the usual 0.05. To evaluate the strength of each effect partial eta squared values were computed for all ANOVA factors. This index reflects the proportion of variance attributable to each factor.

The formulation of the biodynamic calendar for wine drinkers’ hypothesis [5] proposes that the changes in sensory experience between Fruit and Root days affect the sensory experience of wines in general. To evaluate whether there was any interaction between specific wines and the biodynamic tasting days (Fruit vs Root day) we carried out a second series of mixed effects with wine as a fixed factor.

Finally, to specifically explore whether there was a difference for the one wine in the sample set that was produced by biodynamic methods, we also calculated ANOVA for only that wine, with condition and replication as fixed factors, subject as random factor, and the condition by replicate interaction.

#### Link between sensory data and wine basic physico-chemical parameters

To obtain a more synthetic picture of the sensory data and evaluate their link with basic physico-chemical parameters, we carried out a principal component analysis (PCA). The sensory descriptors were entered into the analysis as active variables. Each wine was represented by four rows corresponding to the four tastings of each wine (two tastings on each tasting day). The physico-chemical parameters were entered as supplementary, continuous variables. The type of tasting day (Fruit vs Root) and wine origin were entered as supplementary nominal variables to evaluate whether these two factors influenced the global description of the wines.

## Results

### Post-experiment questionnaire data

Eighteen of the 19 study participants returned completed questionnaires. Ten of the 18 tasters reported knowledge of the existence of the biodynamic wine drinkers’ calendar, while eight had no knowledge of it. Of the 10 tasters aware of the calendar’s existence, three reported ever having tasted wine according to the instructions of the calendar, and one person only reported previously tasting wines according to the lunar cycle. Interestingly, two of the three who reported previously having tasted wines according to the calendar also reported in a prior question that they had never tasted wines “according to the lunar cycle (the stars and the moon)”. Hence, an inconsistency occurs, suggesting some ignorance or ambiguity for these individuals regarding the basis for the instructions in the calendar. Finally, when asked in the final question about the current tasting of Pinot noir wines, not one person commented that they had tasted the wines according to either the lunar cycle or the calendar.

### Sensory data

**Fig 1:**
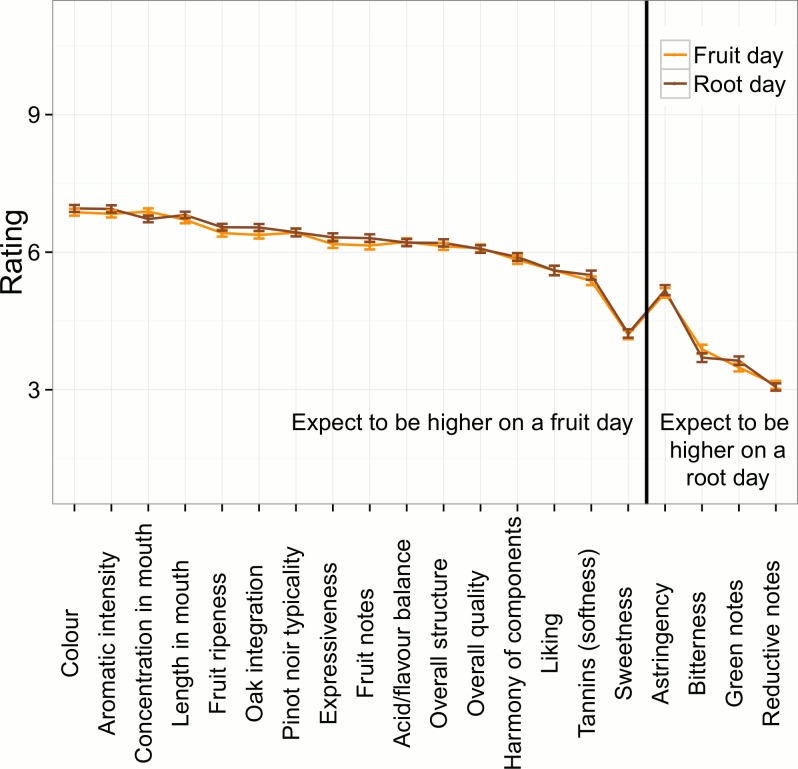
Mean ratings by Fruit/Root day. Mean ratings (+/- SE) to the twelve wines for each descriptor on Fruit days and on Root days.

Mean ratings to the 12 wines for each descriptor on Fruit days and on Root days are presented in Fig 1. The ANOVA results, with wine as random factor, are summarised in Table 3. There was a significant effect (p < 0.10) for three descriptors, bitterness, oak integration, and concentration. However, one only of these was consistent with the biodynamic wine tasting calendar prediction, with concentration in mouth being higher on a Fruit than a Root day. For the other two significant effects, oak integration was higher on a Root day and bitterness was higher on a Fruit day, these two effects being counter to the biodynamic calendar prediction and anecdotal evidence that the wines would taste “better” on a Fruit than Root day. For many of the other wine characteristics that could be expected to be rated higher on a Fruit day (left of Fig 1), responses were in fact slightly higher on Root days. Similarly, descriptors that could be expected to be rated lower on a Fruit day were rated higher on a Fruit day (right of Fig 1). Further supporting the lack of evidence for superior perceived wine quality on a Fruit day in comparison with a Root day, partial eta squared values did not exceed 0.005, meaning the differences in ratings between a Fruit and a Root day tasting accounted for less than half a percent of variation.

**Table 3:** Summary of ANOVA analysis for each descriptor.

Descriptor	Effect				Variance explained			
	Condition^a^	Replicate^b^	Condition *Replicate	Wine	Condition	Replicate	Condition *Replicate	Wine
	F(1,877)	F(1,877)	F(1,877)	F(11,877)	(%)	(%)	(%)	(%)
Intensity descriptors								
Aromatic intensity	1.4	1.7	0.1	13.4***	0	0	0	14
Fruit notes	2.6	0	0.2	18.2***	0	0	0	19
Green notes	2.2	5.3*	1.5	4.0***	0	1	0	5
Reductive notes	0.3	0.6	0.1	10.5***	0	0	0	12
Concentration in mouth	3.6†	0.3	5.1*	7.4***	0	0	1	9
Bitterness	3.1†	5.6*	0.2	4.0***	0	1	0	5
Astringency	0.3	5.7*	0.4	20.3***	0	1	0	20
Sweetness	0.1	18.9***	1.6	6.9***	0	2	0	8
Quality evaluation								
Overall quality	0	0.4	1	12.5***	0	0	0	14
Oak integration	2.9†	3.7†	0.5	13.2***	0	0	0	7
Acid/flavour balance	0	2.2	0.1	6.3***	0	0	0	7
Harmony of components	0.3	3.1†	0	11.4***	0	0	0	13
Overall structure	0.6	3.1†	0.6	9.2***	0	0	0	8
Length in mouth	1.4	1.2	3.1†	19.8***	0	0	0	4
Qualitative descriptors								
Expressiveness	1.7	2.9†	0.1	8.6***	0	0	0	10
Fruit ripeness	1.7	0	2.4	6.0***	0	0	0	7
Tannins	1.4	10.7***	2.2	18.2***	0	1	0	19
Colour	1.3	0.2	1.6	58.0***	0	0	0	40
Overall appreciation								
Pinot noir typicality	0	0	6.9**	5.3***	0	0	1	6
Liking	0	1.3	0.4	10.2***	0	0	0	11
Mean (F/Effect Size)	1.245	3.345	1.405	13.17	0	0.3	0.1	11.9

Note

† p < 0.10.

* p < 0.05.

** p < 0.01.

*** p < 0.001.

^a^ Condition = Fruit vs Root Day.

^b^ Replicate = within-session tastings.

**Fig 2:**
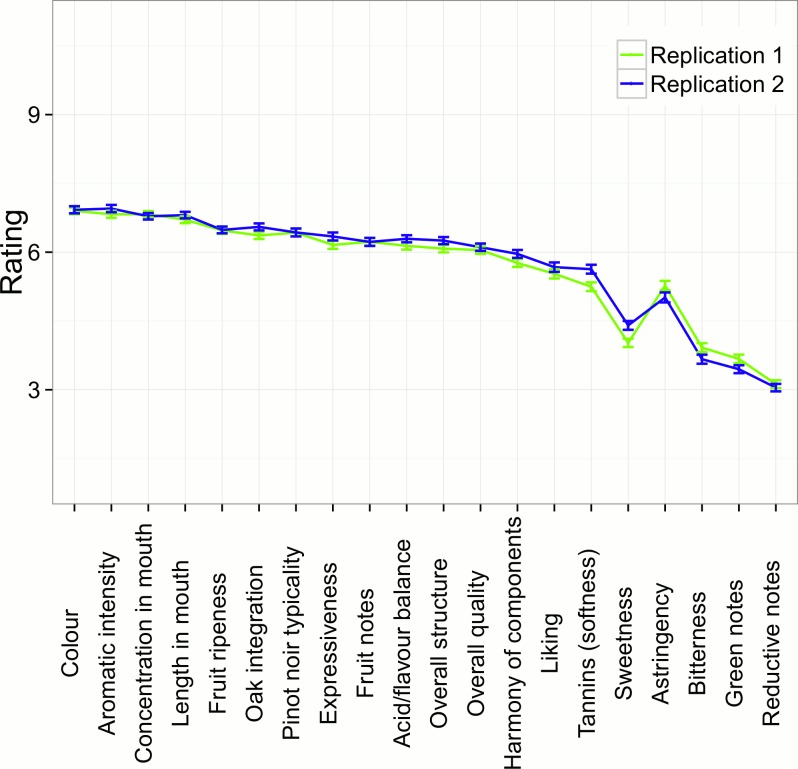
Mean ratings for each sensory descriptor by Replicate 1/Replicate 2. Mean ratings (+/- SE) to the twenty wine descriptors as a function of within-session replicate: Replicate 1 = first assessment of the wines; Replicate 2 = repeat assessment of the wines.

In contrast with the lack of descriptor-rating differences between Fruit and Root days, there were several significant differences between replicate ratings within a session (Table 3; Fig 2). Differences were observed for green notes, bitterness, astringency, sweetness, tannins, oak integration, harmony of components, overall structure and expressiveness. The characteristics that could be considered not desirable in Pinot noir wines [16], namely green notes, bitterness and astringency, were rated higher in the second tasting within a session. Conversely, several qualities desirable in Pinot noir wines including tannin softness, harmony of components, and wine overall expressiveness were judged less positively in the replicate tasting within a session. However, despite these differences being statistically significant, they typically accounted for a small 1% of total variance observed. There were also two statistically significant interactions between replicate and condition, one for Pinot noir typicality and the other for concentration in mouth. However these interactions are not in keeping with the biodynamic wine tasting hypothesis.

In contrast to the small differences attributable to condition of Fruit day versus Root day and to within-session replicate tasting, a significant effect of Wine, accounting on average for 12% of the variance, was observed for all descriptors [see S1–S3 Figs]. That is, the Pinot noir wines were judged as differing in the varietal and quality wine characteristics measured, including in perceived overall quality and in liking. These results are in agreement with previously published work involving NZ Pinot noir wines [16].

The ANOVA carried out with wine as fixed factor showed no significant wine*condition effects, *p* > .26. [See S1 Fig for detailed results and ratings of each wine for each descriptor by Fruit and Root days].

Analyses for the sole biodynamic wine, wine QRPN, also showed no evidence of differences between Fruit and Root days (*p* > .20). Finally, the data presented in S1 Fig show that type of wine production management (see Table 1), namely conventional, organic or biodynamic, was not a factor in quality ratings of the Pinot noir wines. This last comment should be qualified in that the small sample set of wines in this study included one biodynamic wine only.

### Association between sensory data and wine basic physico-chemical parameters

Mean results of the Winescan^TM^ FT2 (FOSS) analysis on each wine on a Fruit day and a Root day are presented in Table 4. The variability across Fruit and Root day measures, and the negative values for malic acid and glucose, can be interpreted in terms of calibration and measurement error as reported in Foss Application Notes 7a, 9, 11, 13, & 14a [20]. Of importance, there is no evidence that wine composition factors that have potential to influence perception of wine quality (e.g., pH; volatile acidity) changed systematically in the direction predicted by the biodynamic wine drinkers’ calendar.

**Table 4:** Mean data for each wine from Winescan analysis of basic physico-chemical parameters on each of a Fruit Day and a Root Day.

Wine	Day	Reducing sugar	pH	Total Acidity	Tartaric Acid	Malic Acid	Lactic Acid	Ethanol V/V	CO_2_	Volatile Acidity	Glucose	Fructose	Folin C index
LDHR	Fruit	1.0	3.60	3.7	2.2	-0.5	1.4	13.58	366.79	0.7	-0.9	-0.5	54.5
	Root	1.1	3.59	3.7	2.2	-0.5	1.5	13.73	386.93	0.7	-0.8	-0.4	54.2
ASB	Fruit	0.4	3.53	3.6	2.7	-0.3	1.6	12.99	363.61	0.6	-0.6	-1.0	57.6
	Root	0.4	3.53	3.7	2.7	-0.2	1.7	13.15	381.96	0.6	-0.5	-1.0	57.8
CHPN	Fruit	0.1	3.53	3.4	2.4	-0.3	1.4	13.68	421.64	0.5	-0.9	-0.6	58.4
	Root	0.1	3.52	3.4	2.4	-0.2	1.4	13.89	438.34	0.5	-0.8	-0.6	58.8
HPN	Fruit	0.2	3.74	3.5	1.6	-0.4	2.6	13.30	362.05	0.6	-1.8	-0.7	45.1
	Root	0.2	3.74	3.5	1.6	-0.3	2.6	13.49	376.88	0.6	-1.7	-0.7	45.2
MDPN	Fruit	-0.2	3.66	3.5	1.9	-0.3	1.9	13.44	339.47	0.6	-1.7	-1.0	62.5
	Root	-0.1	3.67	3.5	1.9	-0.3	1.9	13.61	359.89	0.7	-1.5	-1.0	63.4
APN	Fruit	0.1	3.56	3.9	2.2	-0.3	2.0	14.05	416.58	0.7	-0.8	-0.8	57.3
	Root	0.1	3.57	3.9	2.2	-0.2	2.0	14.26	429.71	0.7	-0.7	-0.8	58.3
QRPN	Fruit	-0.1	3.61	3.4	1.8	-0.2	1.7	13.99	466.95	0.5	-1.1	-0.8	56.6
	Root	-0.1	3.61	3.3	1.8	-0.1	1.7	14.21	502.30	0.5	-1.0	-0.7	57.5
WWW	Fruit	0.9	3.69	3.7	2.3	-0.2	1.7	14.07	389.12	0.7	-0.4	-0.4	58.3
	Root	0.8	3.69	3.7	2.3	-0.2	1.6	14.25	410.90	0.7	-0.4	-0.4	59.2
PPPN	Fruit	1.2	3.55	3.6	2.4	-0.5	1.7	12.72	387.64	0.5	-0.1	-0.5	51.3
	Root	1.1	3.54	3.6	2.3	-0.4	1.7	12.99	419.57	0.5	-0.2	-0.5	52.2
MVTT	Fruit	0.5	3.61	3.4	2.4	-0.2	1.3	13.82	447.40	0.6	-0.6	-0.6	56.3
	Root	0.4	3.61	3.4	2.4	-0.1	1.4	14.03	464.70	0.6	-0.5	-0.7	56.5
WNPN	Fruit	0.6	3.62	3.3	1.6	-0.3	1.9	13.07	436.04	0.5	-0.7	-0.7	54.1
	Root	0.4	3.63	3.4	1.6	-0.2	1.9	13.28	460.76	0.5	-0.7	-0.7	54.1
NMPN	Fruit	0.5	3.64	3.6	1.9	-0.2	1.7	13.12	364.19	0.6	-0.7	-0.7	56.5
	Root	0.4	3.65	3.5	1.8	-0.1	1.7	13.37	386.93	0.6	-0.7	-0.6	56.9

**Fig 3:**
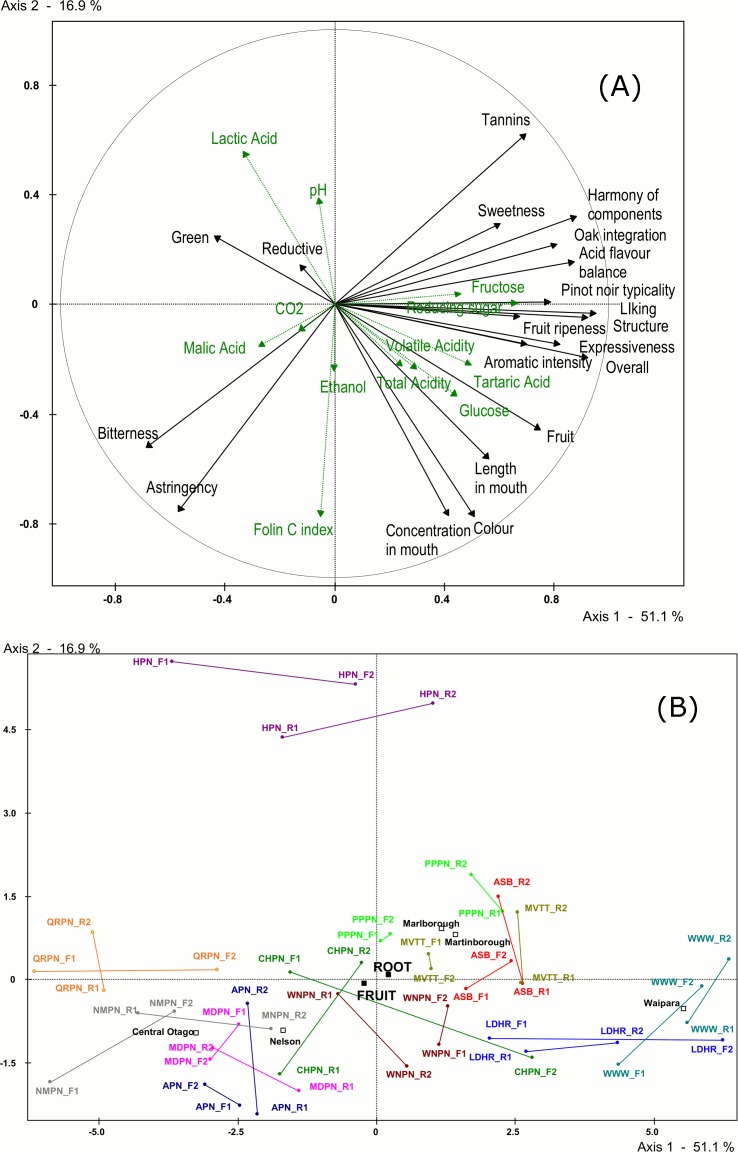
PCA of sensory data (active variable), wine basic parameters (supplementary continuous variables), tasting days and wine origin (supplementary nominal variable). PCA of sensory data: (A) Loadings of sensory variables (black) and basic physico-chemical variables (green) plotted in PCA space; (B) Projections of each wine by Fruit/Root day and replicate 1 and 2 in PCA space, along with centroids for region (Central Otago, Marlborough, Martinborough, Nelson, Wairarapa) and centroids for Fruit/Root days.

PCA results are reported in Fig 3. The first principal component (51% of variance) opposes the wines that were the most liked, these exhibiting ripe fruits, intense aroma, harmony, and expressiveness, with wines more green, astringent, and bitter (Fig 3A). These latter two sensory characteristics were associated with several wine composition parameters, namely Folin C Index (a measure of wine total phenolics), malic acid, ethanol, and CO_2_. On the other hand, more positive wine attributes such as fruit ripeness, concentration, expressiveness, good structure, acid-flavour balance, harmony, sweetness, and soft tannins were associated with wine composition factors related to sugars and acids, including volatile acidity.

According to our experimental hypothesis based on the lunar calendar [5], wines tasted on Fruit days should have high loading on the positive side of this component and wine tasted on Root days should load on the negative side. Fig 3B shows that there is no evidence for this as there are as many wines tasted on the Root as on the Fruit days projecting onto both sides of the component. The second principal component (17% of variance) opposes one wine to all other wines independently of the type of day on which it was tasted. Globally, the position of the barycentres of the nominal variables on Fig 3B shows no effect of tasting day (the Fruit and Root day barycentres project very close to one another in the centre of the map) but shows an effect of wine origin (the barycentres of origins are spread out along the first component). This last result indicates that the absence of an effect of tasting day cannot be attributed to a lack of sensitivity of the participants given that their descriptions were sensitive enough to detect differences in terms of wine origin. This is in agreement with the ANOVA results that show that the differences between repeat tastings *within* a tasting day were of the same magnitude as the differences *between* tasting days.

### Meteorological information

Table 5 shows meteorological data and Table 6 reports measures of air pressure (hPa) for Blenheim, the location of the study, during each tasting session. Although there were differences in some measures, there is no systematic difference across Fruit and Root days that could conceivably be responsible for the minor differences in either sensory or physico-chemical data reported on the 12 wines.

**Table 5:** Meteorological data collected at Blenheim Weather Station, Grovetown Park campus of the Marlborough Research Centre, Blenheim, New Zealand, between the hours of 12 md and 7 pm during which time the experimental wine tastings took place.

Date	Day	Mean dry bulb temperature °C	Mean sunshine (minutes)	Total Rainfall (mm)	Mean wind Speed (Km/hour)	Mean relative humidity (%)
25/11/14	FRUIT	21.54	31	0	16.55	64.47
26/11/14	FRUIT	24.95	480	0	28.39	29.08
27/11/14	ROOT	21.95	411	0	26.87	44.3
4/12/14	FRUIT	22.39	81	0	18.69	50.01
5/12/14	ROOT	14.71	0	0	8.93	78.7

**Table 6:** Blenheim hourly air pressure readings taken from the NZ Meteorological web site during conduction of the tastings.

Date	Time	Air pressure hPa	Date	Time	Air pressure hPa
25/11/14	1pm	1009	4/12/14	1pm	1014
FRUIT DAY	2pm	1010	FRUIT DAY	2pm	1014
	3pm	1008		3pm	1014
	4pm	1007		4pm	1013
	5pm	1006		5pm	1014
	6pm	1005		6pm	1014
	7pm	1005		7pm	1014
26/11/14	1pm	1003	5/12/14	1pm	1022
FRUIT DAY	2pm	1003	ROOT DAY	2pm	1022
	3pm	1003		3pm	1021
	4pm	1003		4pm	1022
	5pm	1003		5pm	1022
	6pm	1003		6pm	1022
	7pm	1003		7pm	1022
27/11/14	1pm	1003			
ROOT DAY	2pm	1002			
	3pm	1001			
	4pm	1001			
	5pm	1001			
	6pm	1000			
	7pm	1001			

## Discussion

The aim of the present study was to test an extension of biodynamic agriculture which argues that wines will taste different in systematic ways on days designated by the biodynamic calendar for wine drinkers [5] as favourable (Fruit days) or unfavourable (Root days) for wine tasting. The outcome of the study is clear; the results demonstrate that judgments to the twelve Pinot noir wines were little influenced by tasting day. That is, there was little variability in descriptor ratings by experienced wine professionals between tastings of the wines on Fruit days versus Root days, despite our hypothesis testing adopting a lenient criterion for rejection of the null hypothesis. In fact, there was more variability within session, where wines were tasted twice on either a Fruit day or a Root day, than between sessions (Fruit vs Root day). As well, there were significant differences amongst the Pinot noir wines reported for every wine characteristic on which the wines were evaluated. These latter effects argue that failure to support our experimental hypothesis was not due to lack of discrimination by our experienced, wine-professional tasters who clearly found and reported significant differences amongst the twelve wines including in overall quality.

To address the topic under investigation, we implemented various essential methodological requirements, the majority of which presumably are lacking when wine industry tastings are conducted according to the biodynamic wine drinkers’ calendar [12] and when positive results are reported in wine industry media. The most important of these was to minimise confounding variables (e.g., by retaining the same order for the 12 wines for each taster across their four evaluations of the wines) and to ensure that the study’s tasters were blind as to the purpose of the study. That is, we needed to know that the tasters did not apply the biodynamic wine drinkers’ calendar [5] guidelines to their tasting experience, this likely influencing their expectations and tasting behaviour during their evaluation of the Pinot noir wines in the current study. That the tasters were blind in terms of not considering the lunar cycle predictions while undertaking the experimental tastings was validated by the post-experiment, questionnaire data. The questionnaire data further informed us as to the validity of our assumption that conducting the study in the Southern Hemisphere would minimise the likelihood that our tasters were current advocates of biodynamic wine tasting practice. Three tasters only reported ever having tasted wine according to the instructions of the calendar, and almost half of the study’s participants were blind to the existence of the wine drinkers’ calendar.

We considered the topic of lunar influence on wine tasting important to investigate for several reasons. First, it is now widely established that contextual factors, both intrinsic and extrinsic, may influence sensorial assessment of wine quality [21], presumably as a result of cognitive influence based on expectations. Suggestions of a lunar effect, widely dismissed by many as pseudo-science or “absolute rubbish” [14], deserved to be tested empirically. Second, there appears increasing reference to wine tasting driven by the lunar calendar in wine industry media [3] [10] [14] and more recently the development of an iPhone App produced by Floris Books. Again, testing the underlying basis for a lunar-effect notion appeared a responsible undertaking given apparent interest in the phenomenon. Third, and related to the second point, a scientific basis for this empirically testable aspect of biodynamic practice could aid scientific respectability for advocates and practitioners of biodynamic philosophy of which there are many in the international wine industry.

Interestingly, our data show that the type of wine production management, namely conventional, organic or biodynamic, was not a factor in determining the influence of Fruit and Root days on how a wine was evaluated. This is in keeping with information provided in the biodynamic wine drinkers’ calendar [5]. Further, and somewhat as a side issue, type of wine production was not a factor in overall quality ratings of the Pinot noir wines. In fact, several of the wines judged highest in overall quality were wines produced by conventional production practices. These data however must be treated with caution due to the low and unequal numbers of wines from each wine-production category, notably from biodynamic production. A future study, aimed specifically at testing the interaction between type of wine production and tasting day, is required before firm conclusions can be drawn on this point.

While failing to support the major tenet of the biodynamic wine drinkers’ calendar that wines are perceived as tasting better or worse according to the lunar cycle, the question remains as to why some wines can appear to taste better on some days in comparison with others [3]. In the present study we were not able to measure all possible factors pertaining to the taster (e.g., mood or stress level of the taster; influence of ovarian hormones). We did however record data regarding objective measures pertaining to the tasting location that have been put forward by some authors [13] [17] as possibilities for influencing how a wine tastes, namely meteorological and air pressure data. These data provide no evidence of conditions that could lead to systematic influence on how the wines were perceived.

Although the biodynamic wine drinkers’ calendar does not comment on whether the proposed source of change in a wine across Fruit and Root days involves perceived differences or differences in wine composition, we conducted basic physico-chemical analysis on the wines in the sample set on each type of tasting day to consider both possibilities. As well, we sourced all the wines employed in the study from their producers rather than retailers to minimise any bottle differences, and stored the wines in the same location prior to the experiment being conducted. Information from the developers of Foss Winescan methodology demonstrates that any differences in our data demonstrate variability within a range expected due to calibration and measurement error [20].

## Conclusion

In conclusion, the findings reported in the present study provide no evidence in support of the notion that how a wine tastes is associated with the lunar cycle. The Pinot noir wines in the sample set were judged by experienced wine professionals as varying significantly in a range of characteristics. However, the day on which there were tasted did not influence these judgments. It is conceivable that the anecdotal reports of sensory effects that have been described in wine-industry media could be due to expectation effects rather than actual differences in the wines. Consumers expecting a wine to be more expressive and aromatic on Fruit days might actually perceive them as such through top down cognitive effects [22]. Such top down effects involving a range of factors have been reported previously. For example, Rose Pangborn and colleagues found that a white wine colored pink to give it the appearance of Rosé wine was perceived by wine professionals as sweeter than a non-coloured wine sample [23]. Likewise, researchers in Bordeaux reported that colouring a white wine with odourless anthocyanin to make it red led wine experts to describe the wine’s flavour as that of a red wine [24]. These results highlight the importance of testing, where possible, anecdotally-based notions and practices in the food and beverage industries. Further work, replicating this study and manipulating the lunar calendar information provided to the tasters, may help in validating the hypothesis pertaining to expectation-driven effects.

## Supporting Information

S1 Fig Mean descriptor rating for each descriptor and each wine by condition (fruit day, root day). (DOCX)

S2 Fig Mean descriptor rating for each descriptor and each wine by replicate within fruit day session. (DOCX)

S3 Fig Mean descriptor rating for each descriptor and each wine by replicate within root day session. (DOCX)

S1 Questionnaire Questionnaire completed by each participant at the end of the experiment. (DOCX)
