# Supplementary material for: Expectation or Sensorial Reality? An Empirical Investigation of the Biodynamic Calendar for Wine Drinkers
Source: PLoS One. 2017 Jan 3;12(1):e0169257. doi: 10.1371/journal.pone.0169257 (PMC5207694; doi:10.1371/journal.pone.0169257)
Supplement: S3 Fig — (DOCX) [file pone.0169257.s003.docx]

**S3 Fig. Mean descriptor rating for each descriptor and each wine by replicate within root day session**
