# Supplementary material for: Expectation or Sensorial Reality? An Empirical Investigation of the Biodynamic Calendar for Wine Drinkers
Source: PLoS One. 2017 Jan 3;12(1):e0169257. doi: 10.1371/journal.pone.0169257 (PMC5207694; doi:10.1371/journal.pone.0169257)
Supplement: S1 Questionnaire — (DOCX) [file pone.0169257.s004.docx]

**S1 File.** **Questionnaire completed by each participant at the end of the experiment.**

**CONFIDENTIAL**

**Short questionnaire for Pinot noir study tasters to complete**

Please answer with honesty the questions below. There are no right or wrong answers. We are interested in YOUR ideas.

**What type of wines do you currently taste the most often?**

Rank the wines below from the least frequent (1) to the most frequent (4)

- Sauvignon blanc __________
- Chardonnay __________
- Pinot noir __________
- Cabernet Sauvignon __________

**When you taste wines professionally or for personal pleasure do you ever consider any of the following**? (check the appropriate answers)

- Time of day yes □ no □
- Your mood yes □ no □
- The weather pattern (e.g., sun, rain, wind) yes □ no □
- The lunar cycle (position of stars and moon) yes □ no □

**Have you ever heard of the biodynamic wine drinkers’ calendar?**  yes □ no □

**Have you ever tasted wines according to the instructions in the biodynamic wine drinkers’ calendar?**  yes □ no □

**Do you have any comments you’d like to make on the recent Pinot noir tastings?**

**THANK YOU**
